# Human and Animal Fecal Contamination of Community Water Sources, Stored Drinking Water and Hands in Rural India Measured with Validated Microbial Source Tracking Assays

**DOI:** 10.4269/ajtmh.14-0824

**Published:** 2015-09-02

**Authors:** Alexander Schriewer, Mitsunori Odagiri, Stefan Wuertz, Pravas R. Misra, Pinaki Panigrahi, Thomas Clasen, Marion W. Jenkins

**Affiliations:** Department of Civil and Environmental Engineering, University of California, Davis, California; Singapore Centre on Environmental Life Sciences Engineering, Nanyang Technological University, Singapore; Asian Institute of Public Health, Bhubaneswar, Odisha, India; Department of Epidemiology and Pediatrics, University of Nebraska Medical Center, Omaha, Nebraska; Rollins School of Public Health, Emory University, Atlanta, Georgia; Disease Control Department, Faculty of Infectious and Tropical Diseases, London School of Hygiene and Tropical Medicine, London, United Kingdom

## Abstract

We examined pathways of exposure to fecal contamination of human and animal origin in 24 villages in Odisha, India. In a cross-sectional study during the monsoon season, fecal exposure via community water sources (*N* = 123) and in the home (*N* = 137) was assessed using human- and nonhuman-associated *Bacteroidales* microbial source tracking (MST) markers and fecal coliforms (FCs). Detection rates and marker concentrations were examined to pinpoint pathways of human fecal exposure in the public and domestic domains of disease transmission in study communities. Human fecal markers were detected much more frequently in the domestic domain (45% of households) than in public domain sources (8% of ponds; 4% of groundwater drinking sources). Animal fecal markers were widely detected in both domains (74% of ponds, 96% of households, 10% of groundwater drinking sources), indicating ubiquitous risks of exposure to animal feces and zoonotic pathogens. This study confirms an often suggested contamination link from hands to stored water in the home in developing countries separately for mothers' and children's hands and both human and animal fecal contamination. In contrast to MST markers, FCs provided a poor metric to assess risks of exposure to fecal contamination of human origin in this rural setting.

## Introduction

Diarrhea is a leading cause of child mortality with significant adverse long-term implications for child development.^1^ Water supply, water quality, sanitation, and hygiene (WASH) are fundamental to reducing fecal-oral transmission of enteric pathogens causing much of the global child diarrhea disease burden.^2^ Although WASH interventions can significantly lower diarrhea risk, the performance of individual components in specific settings is highly variable with no effects sometimes observed.^3^ Apart from study methodological and compliance heterogeneity, inconsistent outcomes are also plausible if dominant pathways of endemic transmission differ between study sites. Likewise, where zoonotic pathogens contribute to the endemic diarrhea disease burden through exposure to animal excreta,^4^ conventional WASH interventions focused on exposure to human excreta alone may have limited health impacts.

Examining fecal contamination across multiple fecal-oral transmission pathways and identifying sources of fecal exposure, for example, whether human or animal, can help pinpoint which routes pose risks to human health.^5^ Such understanding can improve selection and design of WASH intervention strategies in a given setting. Exposure to fecal contamination can occur at community scale for example via contaminated public water sources or food supplies or originate within the home from household contaminated hands, fomites, prepared food, or storage of drinking water. Knowing whether human fecal exposure is occurring mainly in the public domain, or mainly in the domestic domain also has implications for the loci of control over prevention, the geographic extent and number of potential source(s) of pathogens, appropriate roles for public policy, and how interventions may need to be designed, delivered, and measured.^6^

Toward this end, we examine and compare exposure to fecal contamination of human and animal origin via multiple public and domestic domain routes of diarrhea pathogen transmission in similar rural coastal communities in Odisha State in India. Because standard fecal indicator bacteria (FIB) (*Escherichia coli*, fecal coliforms (FCs), enterococci) are unable to distinguish host sources of fecal contamination, more sophisticated detection methods are needed in rural India where open defecation is common and occurs without significant spatial separation from domestic animal fecal loading. Quantitative detection of host-associated fecal *Bacteroidales* genetic markers via quantitative real-time polymerase chain reaction (qPCR) to identify contamination sources, referred to as microbial source tracking (MST), is increasingly applied as an alternative or addition to FIB in both developed and developing countries to identify microbial risks emanating from specific fecal sources.^7^ In this study, we applied *Bacteroidales* MST qPCR assays recently validated in India^8^ to measure total, human, and livestock-associated fecal contamination in community surface and groundwater sources, and on hands and in stored drinking water (SDW) in homes. The objectives were to (1) assess pathways and risks of exposure to human and animal fecal pathogens in the public and domestic domains of study villages and (2) discuss implications for diarrhea disease control.

## Materials and Methods

In each village, improved and unimproved community water sources (as defined by the World Health Organization/United Nations Children's Fund Joint Monitoring Program for Water Supply and Sanitation) were sampled, and hand rinses (HRs) and SDW in homes with a child under age five were collected. Samples were analyzed to evaluate levels and sources of fecal contamination in each tested public and domestic domain exposure pathway. Sampling occurred during the monsoon season, a period when diarrhea rates typically rise in the region, between June 19 and July 26, 2012. Prevalence and seasonal trends of reported diarrhea in the study area can be found elsewhere.^9^

### Study communities.

The study comprised 24 villages in Puri District forming a subsample of 100 villages of similar size and socioeconomic characteristics enrolled in a cluster randomized controlled trial of the health impacts of rural sanitation in India.^10^ All trial villages had access to improved drinking water supplies consisting predominantly of public and private tube wells (86% of households) but < 10% sanitation coverage (all improved) before the 2011 latrine intervention (detailed in Clasen and others^10^). Public tube wells mostly drew groundwater from deeper depths than the shallow tube wells installed privately by homeowners. Despite access to improved water sources, the majority of trial households used open ponds daily for nondrinking purposes, such as anal cleansing after defecation, bathing, brushing teeth, laundry, and cleaning utensils. Trial households belonged predominantly to lower castes (57%), qualified as poor (62%), were Hindu (100%) and owned livestock (59%), comprising cattle, sheep, goat, and buffalo, in order of most common species owned. Details of trial villages have been published previously.^10^

### Village and household selection.

Trial intervention villages were paired with the geographically nearest control village, creating 50 proximal pairs from which 12 were randomly selected into this study. In each village, households with a child under five were stratified by the use of a private or public tube well for drinking, and six randomly selected, aiming for half in each stratum. Where a village had three or fewer private tube well child households, all were included and the balance drawn from public tube well users. Similarly, where very few child households used a public tube well, more private tube well users were selected to reach a sample size of six per village. Up to three additional households per stratum were randomly selected as reserves. If the mother or child was absent, the private tube well was not functioning, or drinking water was typically stored but unavailable, the household was replaced with a reserve household. Two households refused to participate. Four to six households were sampled in each village, for a total of 137 households.

### Public domain water source selection.

During reconnaissance visits before sampling, public tube wells and community ponds were surveyed to identify those most heavily used. Our goal was to sample two public tube wells (deep groundwater), two private tube wells (shallow groundwater), and two public ponds (surface water) in each village. Selected public tube wells were those from which enrolled households had drawn their SDW with the addition of another heavily used public tube well when all used the same one. The two most heavily used ponds were selected if more than two existed. Ponds were sampled at the women's access area because young children accompany their mothers. In total, three to six public domain water sources per village were sampled once, comprising 43 public and 41 private tube wells, and 39 ponds.

### Water source sample collection and processing.

Twenty liters were collected for molecular analysis using ten 2-L open mouth plastic bottles (Tarsons, Kolkata, India) thoroughly washed, bleached with 10% sodium hypochlorite solution, and air dried for > 24 hours before use. Each bottle was rinsed multiple times with water from the source before taking the sample. In addition, a 100-mL aliquot was collected in a sterile 4-oz Whirl-Pak (NASCO Corp., Fort Atkinson, WI) for FC measurement. Source samples were collected simultaneously with household samples (see below) between 8 and 11 am during a single morning visit to each village and spatially paired villages were sampling on consecutive days to reduce temporal confounding. Upon collection, samples were placed on ice, transported to the laboratory in Bhubaneswar, and processed within 8 hours of collection. Each 20-L *s*ample was filtered via hollow fiber ultrafiltration using a previously published protocol.^11^ A portion of the filtration retentate (2.5 mL) was mixed with 2.5 mL of RNALater (Life Technologies, Carlsbad, CA) and stored at −70°C until transport back to the University of California, Davis (UCD) for molecular analysis.

### Household SDW and HR collection and processing.

Approximately 500 mL of SDW was collected for molecular analysis and a further 100 mL separately collected for FC measurement by asking the mother to serve drinking water into sterile 69-oz and 4-oz Whirl-Paks, respectively. HRs were collected from the mother and the youngest child following Pickering and others^12^ using a 69-oz Whirl-Pak containing 350 mL of sterile distilled water. Samples for molecular analysis were filtered through 47-mm, 0.45-μm Millipore HA filters (Fisher Scientific, Pittsburg, PA). The entire sample volume was measured and filtered where possible. Some HR samples contained considerable solids resulting in less filtered volume (~100 mL). Each membrane was rolled and placed into a 5-mL cryogenic tube containing 0.5 mL RNALater, vigorously vortexed, and stored at −70°C until transport back to UCD.

### FC measurement.

Samples were tested for FCs within 8 hours of collection using the membrane filtration method^13^ with lauryl sulfate media (Oxoid Limited, Basingstoke, Hampshire, United Kingdom). All 100 mL of a tube well sample was filtered, while two volumes (dilutions) were filtered for other samples, ranging from 100 to 0.25 mL depending on sample turbidity and type. Resultant FC count (colony-forming unit [cfu]) lower and upper detection ranges were: 1–100 cfu/100 mL for tube well samples, 1–40,000 cfu/100 mL for open pond samples, 1–20,000 cfu/100 mL for SDW, and 34–67,200 cfu/2 hands for HR samples. The smaller volume was used when countable results were obtained for both volumes. In analyses of FC concentrations, nondetect (ND) samples (no colonies for either volume) were assigned half the lower detection limit of the larger volume. Too numerous to count samples were assigned 1.5 times the smaller volume upper detection limit.

### Molecular analysis.

Nucleic acids were extracted from retentate samples using the PureLink Viral RNA/DNA Mini Kit (Life Technologies, Carlsbad, CA) and manufacturer protocols, and from filters following Mattioli and others^14^ modified for the Powerwater RNA KIT (MoBio, Carlsbad, CA). Nucleic acid extracts were analyzed via qPCR for total *Bacteroidales*, and for human-, livestock ruminant-, and dog-associated *Bacteroidales* using the BacUni, BacHum, BacCow, and BacCan assays, respectively, following published protocols.^8^

BacCow was shown by Odagiri and others^8^ to be an ideal assay to detect domestic animal fecal sources in India, including all major livestock ruminant species (cow, buffalo, goat, sheep), chicken, and dog. This assay also did not cross-react with any individual human or sewerage fecal samples^8^ and is therefore applied in this study to distinguish fecal sources of domestic and livestock animal origin (henceforth referred to as “animal”) from human origin. BacHum demonstrated 40% sensitivity on human feces from individual hosts, but detected 100% of mixed sewerage samples as human and had the highest accuracy among five human-associated markers tested in India. HumM2, the second best performing assay in the India validation was used on 10% of samples positive for total *Bacteroidales* and negative for BacHum, but did not yield any additional human detection, which would indicate low host source sensitivity in the study context. Because of some observed BacHum marker detection in dog feces in India^8^ and elsewhere,^15^ all human-positive public and domestic domain samples were tested for dog markers using the India-validated BacCan assay and found negative, ruling out false human positives due to dog fecal contamination. BacHum was also shown to cross-react with chicken fecal samples in validation work in India.^8^ However, only 3 of 137 study households owned chickens, thus the possibility of false-positives due to chicken fecal matter can be discounted.

Results are given in gene copies (gc) per milliliter for water samples or per two hands for HRs. Individual sample (lower) limit of detection (SLOD) values were calculated to account for differences in processed sample volumes, for example, of HR samples. To ensure quality control and unbiased estimation of presence/absence detection rates at a standard threshold of detection, for each sample type extreme SLOD outlier samples (> 3σ + the mean SLOD) were removed and the maximum SLOD value of nondetected samples was applied as the detection threshold to classify marker presence/absence. Extremely high SLOD values indicate lost sample volume, very high solids content, or both. In total, two HR samples were extreme SLOD outliers and removed from the data set. Samples with detected markers had statistically significant or nearly so higher SLOD values than samples of the same time without detected markers. As such, assigning the highest SLOD concentration among nondetected samples to the detection threshold ensured unbiased presence/absence assignment with minimum information loss.

For HR samples, SLODs per two hands varied from 325 to 4772 gc (detection threshold concentration: 462 gc), 123 to 1801 gc (614 gc), and 42 to 616 gc (229 gc), respectively, for total (BacUni), human-associated (BacHum), and animal-associated (BacCow) *Bacteroidales* markers. SLOD per milliliter of SDW varied from 0.4 to 5 gc (1 gc), 0.2 to 2 gc (0.6 gc), and 0.1 to 0.6 gc (0.2 gc), whereas tube well and pond SLOD varied from 3 to 20 gc (6 gc), 1 to 8 gc (2 gc), and 0.4 to 3 gc (0.7 gc) per mL, for BacUni, BacHum, and BacCow markers, respectively.

### Licensing of assays.

A research license was obtained from the U.S. Environmental Protection Agency to use the patented HumM2 assay for the duration of our study.

### Statistical analysis.

The χ^2^ test was used for differences in rates of detection of FC and *Bacteroidales* markers between sample types representing different exposure pathways. In analyses of concentrations, FC and MST markers in ND samples were assigned half their SLOD and FC counts (cfu) and *Bacteroidales* marker gene copies were log 10-transformed (referred to as log) before analysis, except for Spearman rank correlation tests of concentrations in domestic domain samples, in which MST marker NDs were treated as zero values. Domestic domain sample types represent different exposure media and have different process volumes and SLOD ranges. When using rank order correlation of two data sets that may contain many NDs, assigning the half SLOD value to NDs causes nondetected samples to be ranked according to their process volumes which would falsely skew test results. Significance was assumed at 0.05 in all analyses. All statistical analyses were performed with SPSS Statistics 22 (SPSS Inc., Chicago, IL).

## Results

### Prevalence of fecal markers in the public domain.

Overall 45–87% of the water sources of each type tested were contaminated with FC, while 44–100% of each type contained detectable levels of *Bacteroidales* (BacUni) (Table 1). As expected, the detection of fecal bacteria was significantly higher in community ponds (87% for FC, 100% for BacUni) than in improved groundwater drinking sources (49% FC, 56% BacUni) (χ^2^, *P* < 0.001 both). Although FC were detected at similar rates in public and private tube wells (45% and 49%, respectively), *Bacteroidales* were detected significantly more frequently in private (shallow) than public (deep) tube wells (68% versus 44%; χ^2^, *P* = 0.026). Detailed prevalence results and statistical tests of differences for public domain sample types are presented in Table 1.

Human-associated fecal markers (BacHum) were found in less than 10% of sampled water sources of any type, with ponds having the highest prevalence (7.7%) and private tube wells the lowest (2.4%). Among positive samples, the geometric mean BacHum concentration (gc/mL) was 15 (*N* = 1), 44 (*N* = 2), and 92 (*N* = 3) for private tube wells, public tube wells, and open ponds, respectively. In 25% of study villages (6/24), at least one community water source was positive for the human marker, with the positive source being an improved drinking water source in half of these villages (3/24; 13%) (Table 1).

Animal fecal markers (BacCow) were detected significantly more frequently in open ponds (75%) than in public (4.7%) or private tube wells (15%). Ponds were more likely to be detected with animal- than human-sourced fecal matter (~10 times more frequently detected), as were private tube wells (~6 times more frequently detected). In public tube wells (deep groundwater), rates of detection of either fecal source marker were equally low. Across study villages, 83% (20/24) had one or more community water source positive for BacCow, with this being an improved drinking source in 21% (5/24) of villages.

### Prevalence of fecal markers in the domestic domain.

Fecal bacteria were detected in every sampled household (100% positive for any marker) (Table 2). *Bacteroidales* were detected in almost all domestic domain samples (97%). Detection on hands (99% of HRs) was higher than in SDW (95% of samples) (χ^2^ *P* = 0.025). In contrast, FC were detected less frequently than *Bacteroidales* in domestic domain samples overall (70%) but at significantly higher rates in SDW (85%) than on hands (63%) (χ^2^, *P* < 0.001).

Human fecal markers were detected in 45% of households (at least one sample positive) (Table 2). Detection on hands (37% of households) was significantly higher than in SDW (20% of households) (χ^2^, *P* = 0.001). There was no difference in rates of detectable human markers on mothers' and children's hands (both 27%). Among positive samples, the geometric mean BacHum concentration (gc/mL or gc/2 hands) for SDW, mothers' HRs, and children's HRs was 4 (*N* = 26), 6,420 (*N* = 37), and 7,650 (*N* = 36), respectively.

Animal markers were detected in nearly every household (96% at least one positive sample). Their detection, as with human fecal markers, was significantly higher on hands (96% of households) than in SDW (52%; χ^2^, *P* ≤ 0.001), and animal marker detection was similarly very high on both mothers' and children's hands (90%).

### Comparing contamination in the domestic and public domains.

**Figure 1.**
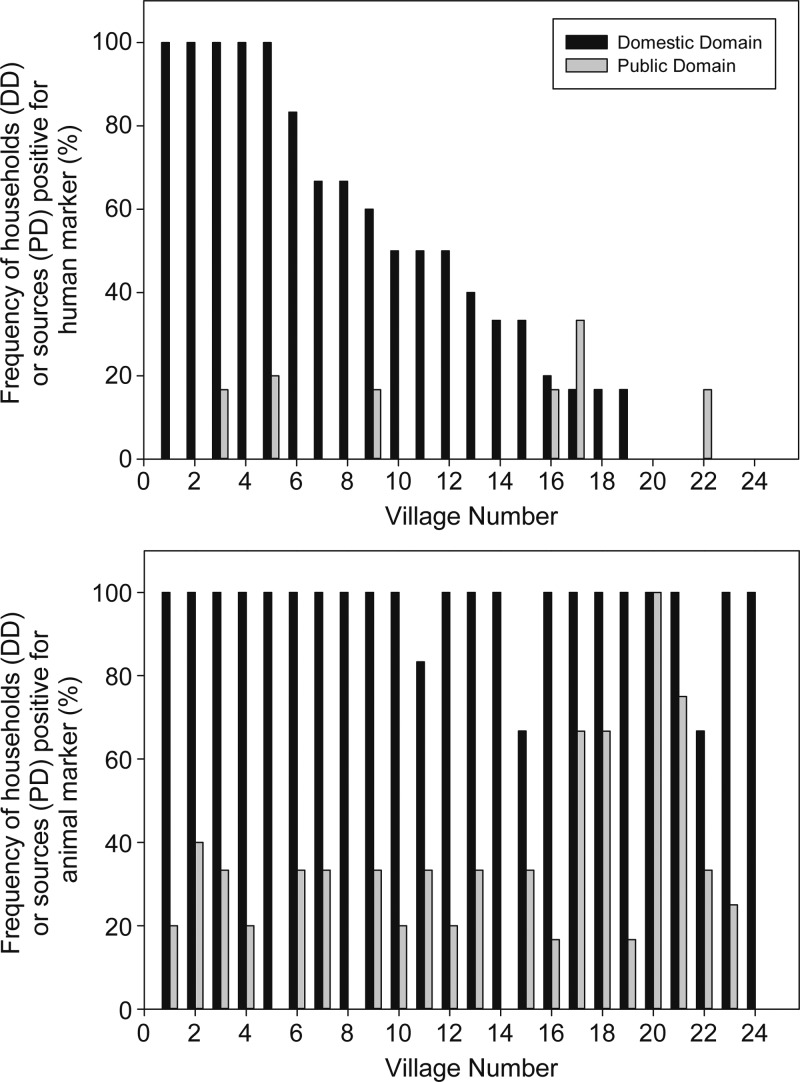
Detection frequency of human (top) and nonhuman (bottom) *Bacteroidales* marker in domestic domain (DD) and public domain (PD) samples from each village, sorted from highest to lowest frequency of domestic domain human marker detection.

Both human and animal markers were detected significantly more frequently in sampled homes (human-positive households: 46%, 95% confidence interval [CI]: 30–62%; animal-positive households: 97%, 95% CI: 92–100% [*N* = 24]) than in sampled community water sources (human-positive sources: 4.5%, 95% CI: 0.5–8.5%; animal-positive sources: 31%, 95% CI: 20–42% [*N* = 24]). Variability across villages in the fraction of sampled households (four to six per village) and community water sources (three to six per village) positive for human fecal markers is shown in Figure 1

(villages plotted from highest to lowest faction of human-positive households). In five villages, every sampled household had at least one human-positive sample, while in five others no human markers were detected at any sampled household. There is no apparent relationship at village level in the likelihood of detecting human fecal contamination in households (the domestic domain) with the likelihood of detecting it in the public domain, as represented by community water sources. In contrast to wide variation in the household rate of detected human fecal contamination across study villages (0–100%), household rates of detected animal fecal contamination were ≥ 67% (≥ 4 positive out of 6) in every village and showed no relationship with household rates of detected human fecal contamination (Figure 1).

### Pathways of contamination in the domestic domain.

**Figure 2.**
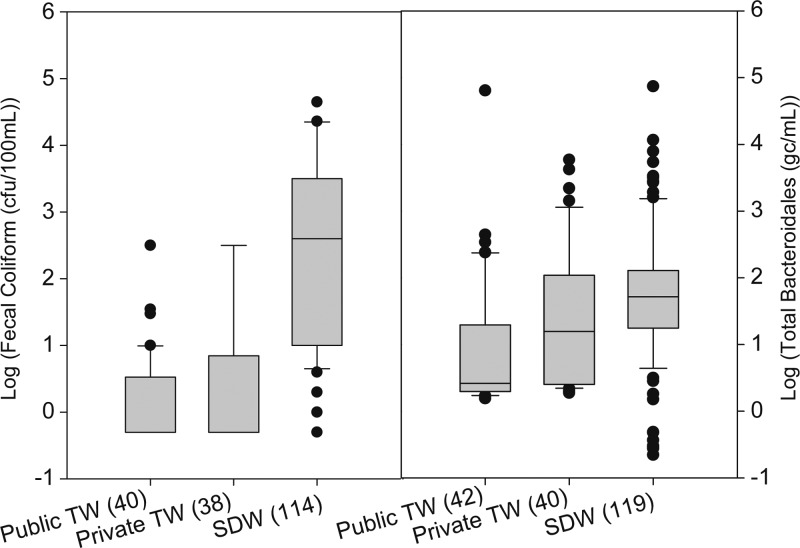
Concentration of fecal coliforms (left) and total *Bacteroidales* markers (right) in improved sources (TW = tube wells) and household stored drinking water (SDW) samples from the same improved sources. Upper and lower edges of boxes denote the 75th and 25th percentiles. Upper and lower bars are the 90th and 10th percentiles, with outliers represented by filled circles. Numbers in parenthesis denote the number of measured samples. Values for samples below or above lower or upper detection limits were replaced with values based on upper and lower detection limits (see statistical analysis section).

Comparing drinking water at the source to SDW in the home, we found significantly lower total *Bacteroidales* marker and FC concentrations in both public and private tube wells than in SDW collected from these same sources on the same day (Figure 2

). Within the home, we found significant positive correlation between contamination levels in SDW and on mothers' hands for each MST marker, ranging from ρ = 0.19 for human markers to ρ = 0.37 for animal markers (Table 3). Significant positive correlations were also found between SDW and children's hands for human (ρ = 0.20) and animal (ρ = 0.24) markers. The strongest positive correlations of fecal contamination between different pathways in the domestic domain were between mothers' and children's hands in the amount of human (ρ = 0.54) and animal (ρ = 0.51) markers present. No significant correlation in FC contamination between any two pathways was found (Table 3).

### FC versus *Bacteroidales* markers as microbial risk indicators in the domestic domain.

No relationship was found between FC and human fecal marker levels for any domestic domain pathway tested (Table 3). However, significant correlation was found between FC and animal marker levels in SDW (ρ = 0.38) and on mothers' hands (ρ = 0.26), but not on children's hands (Table 3). Correlations among *Bacteroidales* markers were highest between total and animal marker levels on mothers' hands (ρ = 0.69), on children's hands (ρ = 0.66), and in SDW (ρ = 0.56). Significant correlations were also found between total and human marker levels on children's hands (ρ = 0.30) and in SDW (ρ = 0.24), but not on mothers' hands (Table 3).

## Discussion

Both traditional fecal indicators and *Bacteroidales* were frequently found in improved drinking water sources. The detection of either indicator in over 70% of tube wells sampled would indicate that fecal contamination in groundwater may be widespread over the study area. The more common source of fecal contamination in improved drinking water sources appears to be livestock and domestic animals, although for many cases the source could not be identified. Four percent of all tube wells had identified human fecal contamination while 10% showed evidence of animal fecal contamination, indicating microbial risks for human health from improved drinking water sources in the study area. Infiltration of fecally contaminated stormwater especially during periods of heavy rainfall and flooding is a likely cause, supported by the fact that detection frequency and concentration of fecal markers were both highest in open ponds, followed by private tube wells (shallow groundwater), and lowest in public tube wells (deeper groundwater). However, once contaminated, hand pumps can act as a reservoir of fecal indicator bacteria (though not of anaerobic *Bacteroidales*) independently of the quality of the pumped water.^16^ Seepage from household pour-flush latrine pits may also contribute to groundwater contamination, given sometimes very high water tables and extended periods of inundation associated with the monsoon season in the study area.^17^

Total *Bacteroidales* markers were detected on a mother's or child's hands in every home sampled. In 37% of households, fecal markers originating from a human source were detected while in over 96% of households an animal source was detected. These findings indicate significant widespread human fecal contamination in the domestic domain and ubiquitous opportunities for exposure to animal fecal contamination in homes throughout the study area. They point to the need for research and interventions focused on reducing exposures in the domestic domain to human fecal pathogens as well as animal-sourced zoonotic pathogens, such as *Giardia*, *Cryptosporidium*, and possible zoonotic strains of pathogenic *E*. *coli* and rotavirus.^4^,^8^,^18^

SDW from improved sources was detected with human fecal matter and with animal fecal matter in 20% and 52% of households, respectively. These rates are lower than rates detected on mothers' or children's hands, despite our being able to detect much lower marker concentrations for each marker in SDW samples than in HR samples (per milliliter, 290 mL mean HR volume). Drinking water stored in the home from improved sources also exhibited significantly higher concentrations of fecal contamination than were present in the improved water sources it came from in the public domain (Figure 2). These observations and the fact that the amounts of human markers when present on mothers' and children's hands were both more abundant (per milliliter) and significantly correlated with amounts present in SDW together form a set of consistent evidence linking hands to post-collection contamination of SDW in this setting. Similar evidence for the BacCow results linked mothers' and children's hands to the contamination of SDW with animal fecal matter. It has been previously suggested that mothers' contaminated hands play a role in degrading stored water quality based on observations and measurements with FIB.^12^,^14^,^19^,^20^ A recent study in rural Tanzania^21^ found that human fecal marker detection on hands corresponded to an increased risk of SDW contamination. This study confirms this link between hands and stored water in the home in a developing community not only independently for mothers' and children's hands but also separately for both human and animal host sources of fecal contamination.

Overall, community ponds used for hygiene were more contaminated with human fecal markers (8%) than improved community drinking water sources (4%), a trend that was also observed in a study examining drinking water sources in Uganda.^22^ But homes were the most contaminated (45%), suggesting that the domestic domain may be an important domain of exposure to and transmission of human fecal pathogens in this setting. Given evidence for cross-contamination of pathways of transmission within the home from highly contaminated hands of both mothers and children, targeted research to understand how hands become contaminated with human fecal pathogens is needed. In addition, efforts to promote and enable greater hygiene in the home, such as hand washing with soap by mothers and young children after contact with any feces, human or animal, and other protective home hygiene practices and structural improvements, are warranted. Continued use of community ponds for personal and domestic hygiene in the study population, despite widespread access to improved water sources, may also present an ongoing risk of exposure to human and zoonotic fecal pathogens.

Percentages of households with detected human fecal contamination varied widely between villages (Figure 1). In some villages, human fecal contamination was not detected on hands or in stored water in any sampled household while in other villages, human contamination was detected in samples from every tested household. These results suggest rural hot spots where domestic exposure to human-derived fecal pathogens is clustered in space and/or time. We could not, however, observe an association between high rates of human fecal contamination in households with corresponding high rates in water sources in the same village on the same day.

Traditionally, testing for standard FIB is a common practice to assess microbial risk and exposure to human fecal pathogens because it requires fewer resources than testing directly for enteric pathogens. However, FIB originates from both humans and animals, and even from non-fecal sources,^16^,^23^,^24^ which calls into question their value for assessing microbial risk of exposure to human enteric pathogens and complicates their use for the identification of pathways of exposure to human fecal contamination. Not surprisingly, we failed to find a correlation between SDW and hand pathways in the home with FC while finding good evidence of cross-contamination from hand to SDW pathways separately for both human and animal fecal markers. Neither did we find any correlation between FCs and the human-associated *Bacteroidales* markers in this setting. Rather, FC in the domestic domain were highly correlated with animal fecal markers. Application of FCs would therefore provide a poor metric to assess risks of microbial exposure or the effectiveness of WASH interventions in reducing risks arising from human fecal contamination in this rural Indian setting.

The study has several important limitations. Observed rates of human fecal contamination of community water sources and of domestic domain exposure pathways tested, especially hands, may be underestimated, given that the BacHum assay showed 40% sensitivity on individual human fecal samples in India^8^ and the minimum number of gene copies needed for detection. When detected, an expected value minimum detectable quantity of fresh human feces (wet weight) present on hands and in stored water in this setting (extrapolating theoretically from performance testing by Odagiri), corresponds to 3.82⋅10^−1^ mg/2 hands and 3.80⋅10^−4^ mg/mL, respectively. For pathways likely to contain feces from mixtures of humans, the case of community water sources, BacHum sensitivity was shown to be high (detecting 100% of sewerage samples). However, these sources are likely to have quite dilute human fecal contamination (compared with sewerage), which may be below the BacHum marker detection threshold, resulting in underestimated human fecal contamination rates for tested community water sources in our study. Although BacHum specificity in India has been reported to be about 80%,^8^ cross-reactivity from the most likely animal sources (i.e., dog and chicken) in our study was investigated and ruled out, increasing confidence that BacHum detection results reflect true human fecal contamination. Comparisons between sample types within and across domains may also be limited by the different limits of detection for each sample type, which are a result of different sample volumes, sample processes and sample matrix specific attributes, mainly turbidity and PCR inhibitor concentrations. We were also unable to test other important pathways of transmission such as contamination of local food supplies used by households and prepared food in the home, or of soil in the home environment and in fields.^25^,^26^ In fact, food supplies brought into the home may be an overlooked but important source of domestic domain human fecal contamination in this setting that has yet to be investigated. Research in Tanzania found evidence of large increases in fecal contamination of mothers' hands following food preparation.^21^,^27^

This study has shown that the application of fecal *Bacteroidales* MST tools is a feasible and more reliable method to assess the risk of exposure to general and human specific fecal contamination than the standard FIB in the study setting. Moreover, it allowed the identification of major transmission pathways for human fecal pathogens in communities with both widespread open defecation and high rates of domestic animal fecal loading that result in environmental fecal contamination of mixed human and nonhuman origin.

## Figures and Tables

**Table 1:** Detection of fecal markers in public domain community water sources

Category (*n* tested for FC/MST)	Detection* frequencies (%)				
	Fecal coliform	Total (BacUni)	Human (BacHum)	Animal (BacCow)	Any fecal marker
Public TW (41/43)	44.8	44.2	4.7	4.7	70.7
Private TWs (39/41)	48.7	68.3	2.4	14.6	82.1
Open ponds (37/39)	86.5	100.0	7.7	74.4	100.0
All sources (117/123)	60.7	69.9	4.9	30.1	83.8
Improved sources (80/84)	48.8	56.0	3.6	9.5	76.3
Villages (any source) (23/24)	95.7	100.0	25.0	83.3	100.0
Villages (improved source) (23/24)	73.9	95.8	12.5	20.8	100.0
	Significance of differences (χ^2^)				
Public vs. private TW	0.996†	0.026	0.585	0.119	0.213
Public TW vs. open pond	< 0.001	< 0.001	0.565	< 0.001	< 0.001
Private TW vs. open pond	< 0.001	< 0.001	0.281	< 0.001	0.007
Improved (TW) vs. open pond	< 0.001	< 0.001	0.323	< 0.001	0.001

FC = fecal coliform; MST = microbial source tracking; TW = tube wells.

* Detection thresholds for BacUni, BacHum, and BacCow = 3, 8, and 2 in gc/mL for ponds and 6, 2, and 1 in gc/mL for TWs, respectively.

† *P* value < 0.05 is considered significant.

**Table 2:** Detection of fecal markers in domestic domain samples

Household samples (*n* tested for FC/MST)	Detection* frequencies (%)				Any fecal marker
	Fecal coliform	Total (BacUni)	Human (BacHum)	Animal (BacCow)	
SDW (125/130)	84.8	94.6	20.0	51.5	98.5
HR-M (137/136)	69.9	98.5	27.2	89.7	100
HR-C (134/135)	55.2	98.5	26.7	89.6	100
All HRs (275/271)	62.7	98.5	26.9	89.7	100
Household hands (HR-M or HR-C) (137/137)	77.6	100	37.2	96.4	100
Households (SDW, HR-M, or HR-C)* (137/137)	94.9	100	44.5	96.4	100
Villages (any household sample) (24/24)	95.8	100	79.2	100	100
	Significance of differences (χ^2^)				
SDW vs. HR-M	0.019	0.078†	0.167	< 0.001	0.147
SDW vs. HR-C	< 0.001	0.079	0.200	< 0.001	0.150
HR-M vs. HR-C	0.110	0.994	0.920	0.984	na‡
HR-M and HR-C vs. SDW	< 0.001	0.025	0.132	< 0.001	0.041
HR vs. SDW (per household)	0.970	0.001	0.001	< 0.001	0.043

FC = fecal coliform; HR-C = hand rinse children; HR-M = hand rinse mother; MST = microbial source tracking; SDW = stored drinking water.

* Detection thresholds for BacUni, BacHum, and BacCow, =1, 0.6, and 0.2 in gc/mL for SDW samples and 463, 614, and 229 in gc/2 hands for HR samples, respectively.

† *P* value < 0.05 considered nonsignificant.

‡ na = not applicable χ^2^ could not be computed (both 100%).

**Table 3:** Domestic domain correlations of *Bacteroidales* associated fecal markers in household SDW and on HR-M and HR-C sorted from highest to lowest

Variable 1*	Variable 2	No. of households	Spearman correlation	
			ρ	*P* value
Correlation between domestic domain pathways				
HR-M human	HR-C human	129	0.540	< 0.001
HR-M animal	HR-C animal	129	0.511	< 0.001
HR-M animal	SDW animal	129	0.367	< 0.001
HR-M total	HR-C total	129	0.322	< 0.001
HR-M total	SDW total	129	0.271	0.002
HR-C animal	SDW animal	127	0.242	0.006
HR-C human	SDW human	127	0.199	0.025
HR-M human	SDW human	129	0.189	0.031
HR-M fecal coliform	HR-C fecal coliform	128	0.116	0.192
HR-M fecal coliform	SDW fecal coliform	123	0.077	0.396
HR-C total	SDW total	127	0.018	0.842
HR-C fecal coliform	SDW fecal coliform	121	−0.076	0.405
Correlation between FC and *Bacteroidales* markers by pathway				
SDW fecal coliform	SDW total	122	0.379	< 0.001
SDW fecal coliform	SDW animal	122	0.378	< 0.001
HR-M fecal coliform	HR-M animal	131	0.257	0.003
HR-M fecal coliform	HR-M total	131	0.250	0.004
HR-C fecal coliform	HR-C human	128	0.152	0.087
HR-C fecal coliform	HR-C total	128	0.096	0.281
HR-C fecal coliform	HR-C animal	128	0.022	0.801
SDW fecal coliform	SDW human	122	0.000	0.999
HR-M fecal coliform	HR-M human	131	−0.024	0.787
Correlation between total and source-associated *Bacteroidales* by pathway				
HR-M total	HR-M animal	131	0.692	< 0.001
HR-C total	HR-C animal	129	0.659	< 0.001
SDW total	SDW animal	129	0.560	< 0.001
SDW human	SDW animal	129	0.329	< 0.001
HR-C total	HR-C human	129	0.296	0.001
SDW total	SDW human	129	0.241	0.006
HR-C human	HR-C animal	129	0.187	0.034
HR-M human	HR-M animal	131	0.080	0.366
HR-M total	HR-M human	131	0.011	0.898

FC = fecal coliform; HR-C = hand rinse children; HR-M = hand rinse mother; SDW = stored drinking water.

* Total, human, animal: representing total (BacUni), human-associated (BacHum), or nonhuman-associated (BacCow) *Bacteroidales* markers.
